# New Literature

**DOI:** 10.1155/S1463924679000328

**Published:** 1979


computer implementation in mass spectrometry.

The chapters fall naturally into two parts. In the first four chapters the author deals with instrumentation and data acquisition, conversion and reduction, while the remaining chapters include approaches to the interpretation and treatment of this data with a final chapter on quantitative analysis.

Fashions in the most acceptable computer system for a laboratory have changed since they were first introduced into mass spectrometry, passing through the stage when centralised computers were advocated.

The author discusses the merits of different arrangements but uses the on-line dedicated mini-computer as the model system when considering acquisition of data. Such systems are the most commonly adopted at present and are likely to remain so with improving specifications, although microprocessors will undoubtedly influence aspects of design.

The excellent chapter on library search should be of value to many workers interested in finding suitable routines to cope with their own individual needs for library facilities. Obviously no single programme is satisfactory for all problems, and the chapter sets out well the various ways of compiling library files of mass spectra and the merits of different systems for searching these files to match an unknown spectrum. When the library file does not contain the unknown spectrum then there is a need for the library programmes to pick out structurally similar compounds or to go further and attempt to elucidate the structural features from known fragmentation rules and other information. Interpretative programmes are extremely large and the author has conveniently summarised the principles involved. In addition the complexities of pattern recognition algorithms applied to unknown spectra should help most readers to follow the expanding literature in this area.

The control of several parts of the mass spectrometer and the acquisition and processing of data from quantitative analysis can be carried out by computer for reasons of convenience, improved precision and the measurement of often large numbers of samples of the same type. The author has reviewed developments in this area in a way, like most of the text, which will give the reader a broad understanding of the progress and utility of computer application. For this reason this book is a worthwhile addition to the bookshelves of all users of mass spectrometers and data systems.

*A. H. Lawson*

### New Literature

### Pharmaceutical sciences

Technicon Industrial Systems has published a special issue of the Pharmaceutical Sciences Newsletter, of particular interest to methods development laboratories now involved with HPLC technology and with the fast-approaching USPXX dissolution requirements. The special newsletter issue features an article on the analytical and physical chemistry section of Wyeth Laboratories Inc., Radnor, a long-term user of continuous-flow systems. Faced with the need to speed up HPLC assays, Wyeth successfully automated a stability-indicating assay for benzodiazepines, using a fully automated analytical system combining sample treatment with liquid chromatography separation techniques.

Copies of the newsletter can be obtained by writing to Technicon Industrial Systems, Tarrytown, New York 10591, U.S.A.

### Photometer for amino acid analysis

A two-page data sheet is now available for the Dionex Model D-550 Dual Channel Ratio Photometer, a high performance detector for amino acid analysis. The data sheet lists features of the photometer and describes the principles of operation. In addition, a functional diagram illustrates the photometer and its relationship to an amino acid analyser. Also included is a table of applications, a figure showing dynamic range and a list of specifications. The Model D-550 employs a number of novel techniques to expand the linear absorbance range to 3.5 Abs and improve the signal-to-noise ratio.

Copies of the sheet are available from Dionex Corporation, 1228 Titan Way, Sunnyvale, CA 94086, U.S.A.

### Water analysis

The Ionics range of laboratory monitors, the 1200 series, for the analysis of natural and waste waters, available from Techmation, are described in a 6-page booklet available on request. The series includes total oxygen demand, total carbon, total organic carbon and combined total oxygen demand/total organic carbon analysers. All the analysers feature an all ceramic sample injection valve that provides accuracy and a repeatable performance with low maintenance. They are fully automated and simple to operate. Reliable solid state electronics give automatic zeroing prior to each analysis and provide precisely controlled operations. The 1200 series can be interfaced with an auto-sampler: the operator is able to analyse and record the results of up to 200 individual samples.

The booklet is available on request from Techmation Ltd., 58 Edgware Way, Middx., U.K.

### High performance thin-layer chromatography

A 16-page CAMAG bulletin 'HPTLC' contains their new HPTLC developing system, several variations of the circular developing device 'U-Chamber' acc. to R. E. Kaiser, and two quantitative submicroliter sample applicators.

A separate 4-page bulletin presents a new TLC/HPTLC scanner, a photodensitometer that has been designed to scan automatically all tracks of a chromatogram, be it conventional or high-performance TLC, developed linear or circular.

Both bulletins are available free from CAMAG, Homburgerstrasse 24, CH-4132 Muttenz/Schweiz.

### Semiconductor devices

Data I/O have recently published a 32-page booklet entitled 'How to survive in the bipolar PROM, FPLA, PAL, MOS EPROM, diode matrix, PMUX, and gate array Programming Jungle!' The book is a good introduction to the many aspects of programmable semiconductor devices and programming techniques.

The first major section of the book discusses the various semiconductor technologies that are used to produce programmable devices. This is followed by tips on handling MOS devices and a method to assess the effectiveness of the ultra-violet lamp used to erase MOS EPROMs. Having provided the reader with the basic background knowledge, the booklet goes on to explain how the devices are programmed under both manual and automatic computer control. Each process is covered and all the jargon normally found in literature describing programming equipment is explained in simple terms. A section is included which provides details of the range of Data I/O programming machines. These machines cover all programming needs from simple 'one off' programming to batch programming on a production line basis. The final pages of the book list the key features of about 300 programmable devices, together with other equipment needed to program them.

The booklet is available from Microsystem Services, Duke Street, High Wycombe, Bucks., U.K.

### Stepping motors

Unimatic Engineers Ltd., now have available the new catalogue from Sigma Instruments Inc., detailing their complete range of stepping and synchronous motors. The catalogue gives full technical information on the motors and also provides applications advice, performance curves and application considerations. Information on Sigma's range of stepping motor drives and controllers is also included. It provides formulae for the calculation of torque and inertia figures coupled with metric/imperial conversion tables for both torque and rotary inertia.

The catalogue is available, free of charge, from Unimatic Engineers Ltd., Granville Road Works, 122 Granville Road, Cricklewood, London NW2, U.K.

### Pollution monitoring equipment

The latest 16-page catalogue from Techmation describes the range of pollution monitoring instrumentation available from a variety of manufacturers: Meloy, Dohrmann, Axel Johnson, Ionics, Turner Designs, Manning and Spectrametrics.

Instruments are described to monitor sulphur gases, oxides of nitrogen, phosphorus, ozone, hydrocarbons and purgeable halides. Total organic sulphur, chloride and nitrogen monitors and calibration sources are also included.

Water pollution monitoring equipment covers a wide range: total organic carbon, total carbon, total oxygen demand and organic contamination monitors; for physical measurements, fluorometers, nephelometers, flow and level recorders and sampling devices. Elemental analysis by plasma emission spectroscopy completes the range.

Free copies of the catalogue are available from Techmation Ltd., 58 Edgware Way, Edgware, Middx., U.K.

### User guide to GASP

The application of a computer program for the analysis of gamma-ray spectra (GASP) at Harwell is described at a level suitable for users with practical experience of spectrometry, but limited knowledge of computer methods in a document published recently by HMSO, price £1.50. It is designed to help users of GASP, written in Fortran language, to be aware of the facilities available.

'A desktop computer system to control Auger Emission Spectroscopy' is the title of another document, price £1.00, published by HMSO to describe how a simple modular interface has been constructed to allow a Hewlett-Packard 9845 desktop computer to control the data acquisition from an Auger Electron Spectrometer.

Both documents are available from HMSO, PO Box 569, London SE1 9NH, U.K.

### Tool and production aid catalogue

The first edition of the Toolrange catalogue, aimed specifically at the electrical industries and containing a comprehensive range of tools, toolkits and production aids, is now available. A mail order service is provided and it offers users access to many selected items from over 60 British and international manufacturers. The catalogue is divided into twelve major product groups from soldering and desoldering through to welding and brazing.

Further details and copies of the catalogue can be obtained from Toolrange Ltd., Upton Road, Reading, Berks. RG3 4JA, U.K.

### Liquid chromatography catalogue

A 44-page catalogue is available from Laboratory Data Control providing full details of their entire product range. The range provides almost every item of equipment required for high performance liquid chromatography from tubing to a complete chromatograph. The catalogue details the main equipment range with operating specifications and ordering information. It is divided into four main sections; complete chromatographs, complete modular systems, individual modules, valves, columns and accessories. A final section contains applications data and advice.

The catalogue is available from European Office, 52 High Street, Stone, Staffs. ST15 8AR, U.K.

### Calendar

### 1979

**The Applications of Microprocessors: A review lecture by Professor J. H. Westcott**
February 8, London, U.K.
*The Executive Secretary, The Royal Society, 6 Carlton House Terrace, London SW1Y 5AG, U.K.*

**Clinical Laboratory Testing in Europe**
February 12-13, London, U.K.
*Robert S. First, Inc., 405 Lexington Ave., New York, 10017, U.S.A.*

**Use of the Microprocessor as a Component in Measurement and Control Equipment**
February 13-15, Stevenage, Herts., U.K.
*Carole Meads, Sira Institute, South Hill, Chislehurst, Kent, U.K.*

**30th Annual Conference on Analytical Chemistry and Applied Spectroscopy**
March 5-9, Cleveland, U.S.A.
*Mrs. L. Briggs, 437 Donald Road, Pittsburgh, Passadena 15235, U.S.A.*

**Process Analytical Instrumentation**
March 19-23, Coventry, U.K.
*Deputy Secretary, The Institute of Measurement and Control, 20 Peel Street, London W8, U.K.*

**International Conferences on Spectroscopy**
March 29-30, Miami, U.S.A.
*Vijay Mohan Bhatnagar, Alena Enterprises of Canada, P.O. Box 1779, Cornwall, Ontario, Canada K6H 5V7.*

**Kongress der Deutschen Gesellschaft fur Laboratoriumsmedizin**
March 29-April 3, Berlin, Germany.
*Wilhelm Syborg, Kongresshall Berlin, John-Foster-Dulles-Allee 10, 1000 Berlin 21, West Germany.*

**River Pollution Control**
April 9-11, Oxford, U.K.
*Water Research Centre, Stevenage Laboratory, Elder Way, Stevenage, Herts. SG1 1TH, U.K.*

**3rd International Symposium on Capillary Chromatography**
April 30-May 3, Hindelang/Allgau, W. Germany.
*R. E. Kaiser, P.O. Box 1308, D-6702 Bad Durkheim 1, Germany.*

**9th Annual Symposium on the Analytical Chemistry of Pollutants**
May 7-9, Georgia, U.S.A.
*Elaine McGarity, Environmental Protection Agency, Environmental Research Laboratory, College Station Road, Athens, Georgia 30605, U.S.A.*

**Current Developments in the Clinical Applications of HPLC, GC and MS**
May 30-June 1, Harrow, U.K.
*Symposium Secretariat, Division of Clinical Chemistry, Clinical Research Centre, Harrow, Middlesex, U.K.*

**3rd European Congress of Clinical Chemistry**
June 3-8, Brighton, U.K.
*Dr. F. Mitchell, CRC, Northwick Park, London, U.K.*

